# STK38 is a PPARγ-interacting protein promoting adipogenesis

**DOI:** 10.1080/21623945.2021.1980257

**Published:** 2021-10-20

**Authors:** Kun Qian, Daozhan Yu, Weiming Wang, Mengqi Jiang, Rongze Yang, Robert Brown, Da-Wei Gong

**Affiliations:** a Department of Gastrointestinal Surgery, The First Affiliated Hospital of Chongqing Medical University, Chongqing, China; b Division of Endocrinology, Diabetes and Nutrition, Department of Medicine, University of Maryland School of Medicine, Baltimore, USA; c Department of Nutrition and Food Hygiene, School of Public Health, China Medical University, Shenyang, China

**Keywords:** PPARγ, STK38, cofactors and adipocytes

## Abstract

Peroxisome proliferator-activated receptor-γ (PPARγ) is the master regulator of adipogenesis, but knowledge about how PPARγ is regulated at the protein level is very limited. We aimed to identify PPARγ-interacting proteins which modulate PPARγ’s protein levels and transactivating activities in human adipocytes. We expressed Flag-tagged PPARγ in human preadipocytes as bait to capture PPARγ-associated proteins, followed by mass spectroscopy and proteomics analysis, which identified serine/threonine kinase 38 (STK38) as a major PPARγ-associated protein. Protein pulldown studies confirmed this protein–protein interaction in transfected cells, and reporter assays demonstrated that STK38 enhanced PPARγ’s transactivating activities without requiring STK38’s kinase activity. In cell-based assays, STK38 increased PPARγ protein stability, extending PPARγ’s half-life from ~1.08 to 1.95 h. Notably, in human preadipocytes, the overexpression of STK38 enhanced adipogenesis, whereas knockdown impaired the process in a PPARγ-dependent manner. Thus, we discovered that STK38 is a novel PPARγ-cofactor promoting adipogenesis, likely through stabilization of PPARγ

## Introduction

Peroxisome proliferator-activated receptor-γ (PPARγ) is a nuclear transcription factor predominantly expressed in adipose tissue and plays a pivotal role in the regulation of adipogenesis. Of clinical importance, thiazolidinediones (TZDs), a major class of drugs for the treatment of type 2 diabetes (T2D), improve whole-body insulin sensitivity through the activation of PPARγ. However, our knowledge about the regulation of PPARγ at the protein level remains incomplete. PPARγ activation begins with ligand binding and subsequent formation of heterodimers with RXR nuclear receptors. This PPARγ–RXR complex then binds PPAR responsive elements (PPREs) of the DNA sequence and recruits basal transcriptional machinery in order to initiate target gene transcription. This process is regulated by many factors but particularly by cofactor proteins that bind to PPARγ and modulate the strength and specificity of PPARγ-mediated transcription. Thus, the identification of PPARγ-associated cofactors is a well-established approach to elucidate the PPARγ signalling mechanism. As a result, a number of PPARγ coactivators, such as PGC1 [1], PRIP (peroxisome proliferator-activated receptor (PPARγ)-interacting protein) [2], and Tip60 [3] have been identified, and these coactivators typically form a complex with PPARγ and enhance transcription at the promoter level.

We have previously reported that the fusion of the activation domain of MyoD (M3) to PPARγ significantly enhances PPARγ’s transactivating activity, and the resultant ‘super-active’ M3-PPARγ can efficiently promote adipogenesis and convert human myoblasts into brown-like adipocytes [4]. To identify possible cofactors which contribute to either this enhanced M3-PPARγ or unmodified PPARγ’s transacting activities, we performed this study. Here, we report that serine/threonine kinase 38 (STK38) is a novel PPARγ-cofactor that promotes adipogenesis in human preadipocytes.

## Results

### STK38 is a PPARγ-interacting protein

**Figure 1.**
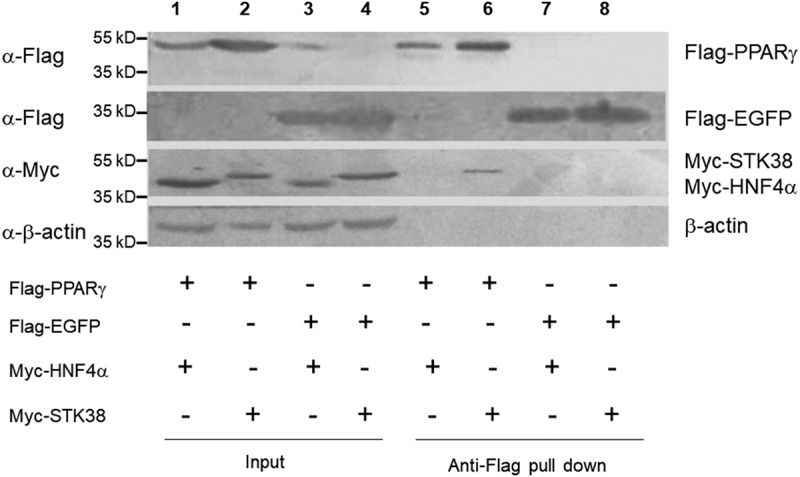
**STK38 is associated with PPARγ**. HEK293 cells were transfected with tagged PPARγ, HNF4α (negative control), EGFP (negative control), or STK38, indicated as plus (+) sign. Cell lysates were precipitated by anti-Flag magnetic beads or by anti-Myc antibody-protein A resin and immunoblotted by antibodies (a) labelled on the left of the blot. Detected proteins are labelled on the right of the blot

We have previously shown that M3-PPARγ is more potent than PPARγ in inducing adipogenesis while retaining its responsiveness to rosiglitazone [4]. To isolate possible PPARγ-binding proteins which may enhance its activity, we conducted proteomics analyses. We infected human preadipocytes with lentiviruses expressing Flag-PPARγ, Flag-M3-PPARγ, and Flag-EGFP and treated the cells with rosiglitazone (Rosi) and insulin. Twenty-four hours post-treatment, we isolated the PPARγ-associated proteins using anti-Flag affinity beads and used the Flag-bound proteins for proteomics analyses. Table 1 shows that a dozen of proteins were identified to bind to Flag-PPARγ and Flag-M3-PPARγ, but not to Flag-EGFP. Chromatography was performed in all three independent experiments. As expected, PPARγ is the most abundant protein isolated from the anti-Flag magnetic beads, given it is Flag-tagged and overexpressed as a bait. Some known PPARγ cofactors such as RXRα, MED1 [5,6], PPM1B [7], and THRAP3 [8] were identified in the experiment, which partially validated that the isolation approach was successful. Notably, a few protein kinases such as serine/threonine-protein kinase 38 (STK38), BTB/POZ domain-containing protein KCTD5, and STK38-like protein (STL38L) were co-eluted with M3-PPARγ and PPARγ. Intriguingly, although there was an overall enrichment of proteins bound to M3-PPARγ, compared to PPARγ, no proteins specific to M3-PPARγ were isolated, suggesting that the enhanced transcriptional activity of M3-PPARγ might not be attributed to a single specific cofactor protein. Nevertheless, these proteins are potentially PPARγ cofactors since they appear specific to PPARγ, but not the M3 domain. As STK38 was the most abundant protein associated with PPARγ and, by virtue of its kinase activity, could be a potential modifier of M3-PPARγ and PPARγ, we focused on this protein and conducted confirmatory immunoprecipitation and functional studies. We next carried out anti-Flag pulldown experiments in HEK293 cells by co-transfecting tagged Myc-STK38 and Flag-PPARγ, and control proteins Flag-EGFP and Myc-HNF4α, a nuclear transcription factor found not to bind PPARγ in a pilot study. As shown in Figure 1, Flag-PPARγ did not pulldown Myc-HNF4α (lane 1 vs. lane5) nor did Flag-EGFP (lane 3 vs. lane 7). Flag-EGFP similarly could not pulldown Myc-STK38 (lane 4 vs. lane 8). However, Myc-STK38 was significantly pulled down by Flag-PPARγ, confirming our proteomics data. This experiment thus demonstrates that PPARγ and STK38 interact with each other.

**Table 1.** List of associated proteins

Proteins	Flag-M3-PPARγ	Flag-PPARγ	Flag-EGFP
**(bound)**	**(#of uni. peps)**	**(#uni. peps)**	**(#uni. peps)**
PPARγ	63	61	0
STK38	31	25	0
PPM1B	17	13	0
RXRα	17	7	0
STK38L	15	4	0
KCTD5	13	15	0
MED1	11	2	0
THRAP3	8	4	0

The number (#: the mean of three independent experiments) of unique peptides (uni. peps) eluted from Flag-M3-PPARγ, Flag-PPARγ, or Flag-EGFP affinity beads. PPM1B: Protein Phosphatase 1B; STK38L: STK38-Like; KCTD5: Potassium Channel Tetramerization Domain Containing 5; MED1: Mediator Subunit 1; THRAP3: Thyroid Hormone Receptor Associated Protein 3.

## STK38 colocalizes with and enhances PPARγ transcriptional activities

**Figure 2.**
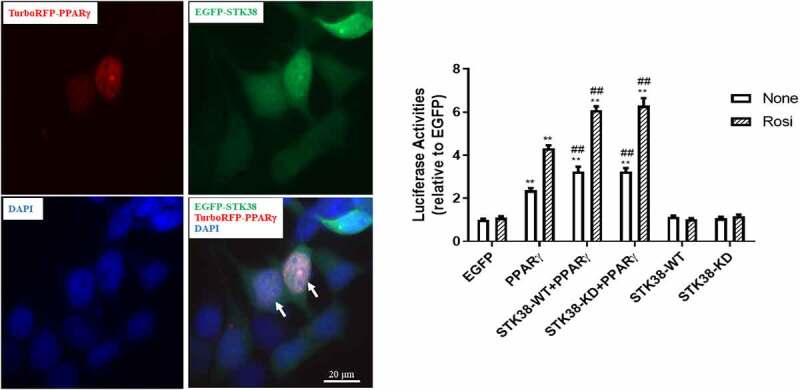
**STK38 colocalizes with PPARγ and enhances its transactivating activities**. (a) Co-localization of STK38 with PPARγ in the nucleus. Plasmids with EGFP-tagged STK38 (EGFP-STK38, green) and turboRFP-tagged PPARγ (turboRFP, red) were co-transfected into HEK293 cells and photographed 48 hours post-transfection under a fluorescence microscope. EGFP-STK38 (green), turboRFP-tagged PPARγ (red), and DAPI (blue). Arrows indicate representative colocalization of EGFP-STK38 with turboRFP in the nucleus in orange. (b) Enhancement of PPARγ-mediated transactivation by STK38. HEK293 cells were transfected with a PPRE reporter and indicated expression vectors and treated with or without rosiglitazone (Rosi). STK38-KD: kinase-dead; STK38-WT: wild-type. Data are mean ± SEM (*n* = 6) **p < 0.01 vs. the corresponding EGFP control; and ##*p* < 0.01 vs. the corresponding PPARγ group

PPARγ is a nuclear protein, whereas STK38’s cellular localization has not been well characterized. To determine whether these two proteins could actually colocalize in the same cell compartment, we co-expressed EGFP-STK38 and turboRFP-PPARγ in HEK293 cells. As depicted in Figure 2a, EGFP-STK38 (green) was present both in the cell nucleus and in the cytoplasm, whereas turboRFP-PPARγ (red) was solely nuclear, and their colocalization (orange) was observed in the nucleus. To investigate the impact of STK38 on PPARγ-mediated transcriptional activities, we next conducted a reporter assay in HEK293 cells wherein a PPRE reporter construct was co-transfected with STK38 variants and PPARγ. As shown in Figure 2b, the reporter activity was significantly increased by PPARγ overexpression, which was further augmented by STK38 co-expression and responsive to rosiglitazone. Interestingly, there was no difference between STK38-WT (Wild-type) vs. STK38-KD (kinase-dead) in the induction of PPARγ activity. Importantly, STK38-WT or STK38-KD alone did not affect reporter activity. This experiment indicates that STK38 increases PPRE reporter activities in a PPARγ-dependent manner and that STK38’s kinase activity is not required for the enhancement.

## STK38 stabilizes PPARγ

**Figure 3.**
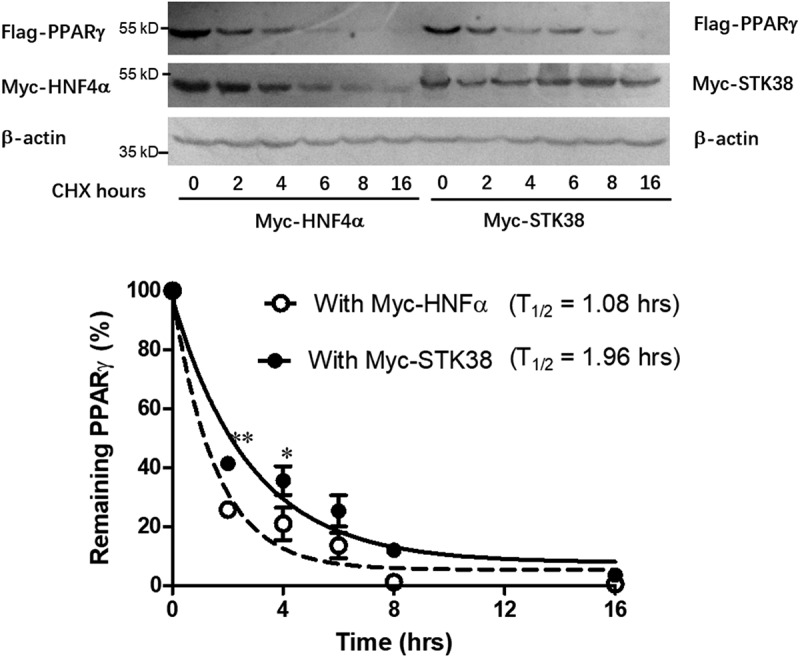
**STK38 stabilizes PPAR**γ ***in vitro***. (a) Stabilization of PPARγ by STK38. HEK293 cells were transfected with Flag-PPARγ and Myc-STK38 or Myc-HNF4α and treated with cycloheximide (CHX) 24 h post-transfection. Cell lysates were collected at the indicated times after the treatment for Western analyses with antibodies (α-) against Flag, Myc, or α-actin. (b) Calculated PPARγ decay when co-expressed with Myc-HNF4α (blank circle) or with Myc-SKT38 (solid circle) with calculated *t*_1/2_. Data are mean ± SEM (*n* = 3 independent studies). *p < 0.05; **p < 0.01

We next investigated the effect of STK38 on PPARγ, protein stability by co-expressing either Myc-tagged STK38 or Myc-HNF4α with Flag-PPARγ, in HEK293 cells and treated the cells with cycloheximide (CHX) to stop protein synthesis. As shown in Figure 3a and 3b, the level of PPARγ decreased with time after the CHX treatment, but its rate of decline was significantly slower when co-expressed with STK38 compared to co-expression with HNF4α. As a result, the estimated half-life (*t*_1/2_ = 1.08 h) of PPARγ without STK38 was extended to T_1/2_ = 1.96 h with STK38. Notably, STK38 protein levels were relatively stable during the 16 h post CHX treatment, as was α-actin. This study illustrates the stabilizing effect of STK38 on PPARγ, resulting in slowed protein degradation of PPARγ.

## STK38 promotes adipogenesis in a PPARγ-dependent manner

**Figure 4.**
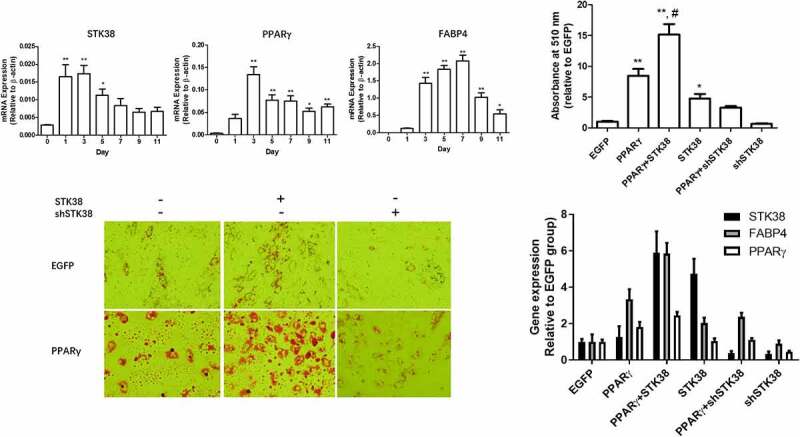
**STK38 promotes adipogenesis**. (a) Time-course of gene expression of STK38, PPARγ, and FABP4 during adipocyte differentiation. Preadipocytes were subjected to adipogenic differentiation, and cells were collected at the indicated times for qPCR analyses. Data are mean ± SEM, *n* = 3, **p < 0.01; *p < 0.05 vs. day 0. (b and c) Oil-Red-O staining and quantification of human preadipocytes infected with PPARγ, EGFP, STK38, and shSTK38 lentiviruses and differentiated for 11 days. Data are mean ± SEM (*n* = 3). **p < 0.01; *P < 0.05 vs. EGFP; #*p* < 0.05 vs. PPARγ. (d) Gene expression analyses of differentiated adipocytes infected with lentiviruses as indicated. Data are expressed as mean ± SEM (*n* = 3)

To investigate the biological function of STK38 in adipocytes, we examined its gene expression during adipocyte differentiation of human preadipocytes (adipose stromal vascular cells). As shown in Figure 4a, STK38 was drastically induced on day 1 after adipogenic induction, remained higher up to day 3, and then decreased gradually, though still above baseline on days 9 and 11 at the end of the differentiation protocol. By comparison, the expression of PPARγ peaked at day 3 and reduced modestly thereafter during the later phase of differentiation. The expression of the adipocyte marker fatty-acid binding protein 4 (FABP4) slowly increased to its peak at day 7 and then decreased gradually. To understand the functional impact of STK38 on adipogenesis, we conducted depletion and overexpression studies in primary human preadipocytes which had been tested to be not amicable to differentiation. The cells were infected with lentiviruses of EGFP (control), shSTK38, or STK38 with or without PPARγ and then subjected to adipogenic differentiation. Cell adiposity was visually evaluated by Oil Red staining (Figure 4b) and quantification (Figure 4c) of Oil-Red-O. As shown in Figure 4b,c, the overexpression or depletion of STK38 alone slightly increased or decreased adipogenesis, respectively. PPARγ overexpression significantly enhanced adipogenesis compared to baseline. Co-expression of STK38 with PPARγ further increased adipogenesis, whereas STK38 knockdown markedly diminished PPARγ-mediated adipogenesis. Concomitant qPCR analyses confirmed the depletion and overexpression of STK38 by lentiviral shRNA knockdown of STK38 and STK38 overexpression, respectively (Figure 4d), while FABP4 expression levels were in agreement with adiposity staining by Oil-red (Figure 4b and 4c). These findings point to a permissive role of STK38 in PPARγ-mediated adipogenesis.

## Discussion

Our initial aim of the study was to identify putative cofactors for M3-PPARγ, a fusion protein which has shown enhanced transcriptional activities (ref). However, we found no qualitative differences in the bound proteins between M3-PPARγ vs. PPARγ. Instead, we found STK38 consistently bound to both M3-PPARγ and PPARγ and functions as a novel PPARγ-interacting protein promoting adipogenesis.

STK38, also called NDR1, belongs to the family of mammalian nuclear Dbf2-related kinases [9] and is implicated in a wide range of cell functions, including proliferation, apoptosis, autophagy, and morphological changes [10]. Substrate phosphorylation is an important part of STK38’s molecular function [11]. For example, STK38 regulates the cell cycle through phosphorylating p21, a cyclin-Cdk inhibitor [12]. STK38 also phosphorylates and activates the nuclear export protein XPO1, escorting the autophagy regulator Beclin1 out of the nucleus to the cytoplasm. This kinase activity is essential for starvation-induced autophagy [13]. In this study, we found that both kinase-active (wild-type) and kinase-dead STK38 proteins stimulated PPARγ-mediated transcriptional activities to a similar degree, suggesting that the kinase activity is not required for STK38-mediated enhancement of PPARγ transcriptional activity. We next demonstrated that STK38 could significantly slow the degradation of PPARγ, but the underlying mechanism is not known at present. Nevertheless, the level of PPARγ protein has been reported to be regulated at multiple levels by SUMOylation and ubiquitination [14,15], ligand-binding [16], and also protein–protein interactions [17], which directly affect its activities. In this study, we found that PPARγ was quickly degraded, whereas STK38 levels remained relatively stable after inhibition of new protein synthesis by cycloheximide [18]. Thus, the association of the stable STK38 with the labile PPARγ may provide the latter with something of a physical shield from protein-degrading enzymes. Our finding is in line with reports that STK38 modulates its partner protein’s stability via protein–protein interaction. For example, the kinase-dead STK-38 interacts with the MYC protein and reduces its degradation rate [19]. In another instance, STK38 interacts with the co-chaperone Bcl2-associated athanogene 3 (BAG3) and inhibits BAG3-mediated autophagy, independent of its kinase activity. Thus, STK38 appears to serve as a chaperone protein regulating the stability of many proteins through protein–protein interaction irrespective of its kinase activity. On the other hand, since both PPARγ and STK38 are implicated in the regulation of cell cycle, proliferation, and apoptosis [20–22], it is possible that PPARγ may exert its function via regulation of STK38’s function.

To determine the physiological relevance of STK38 to adipogenesis, we investigated the gene expression of STK38 and PPARγ during adipogenic differentiation of human preadipocytes. We found that STK38 induction occurred rapidly and peaked between days 1 and 3, whereas PPARγ expression was induced more slowly and peaked at day 3, indicating that STK38 is an early responsive gene to adipogenic induction. Furthermore, knockdown of STK38 impaired, whereas overexpression of STK38 promoted, adipogenesis. Notably, STK38 significantly enhanced PPARγ overexpression-mediated adipogenesis. Thus, we demonstrated that STK38 plays a permissive role in adipogenesis through PPARγ.

It is noteworthy that STK38L, STK38-like protein, was also isolated in initial pulldown experiments, despite lower abundance (Table 1). STK38L shares an 87% identity with STK38 and likely behaves similarly to STK38 in association with stabilizing PPARγ. However, their regulation during adipogenesis and at different adipose depots may differ. Our recent RNAseq analyses of gene expression indicate that the STK38 increased, whereas STK38L decreased during adipogenesis (unpublished data). Further individual and double knockout studies would be needed to address their roles in adipogenesis.

In summary, through proteomics study, we have identified STK38 as a novel PPARγ-coactivator that stabilizes the PPARγ protein. This mechanism is distinct from most known transcriptional coactivators, which act at the promoter level (ref). Our discovery of STK38 as a likely chaperone protein of PPARγ provides a novel insight into PPARγ biology and its regulation.

## Materials and methods

Rosiglitazone was purchased from Alexis Biochemicals (San Diego, CA, USA); protease inhibitor cocktail from Roche Applied Biosciences (Indianapolis, IN, USA); LipoD293 from Signagen Laboratories (Gaithersburg, MD, USA); anti-Flag M2–magnetic beads, anti-Flag antibody, and 4,6-diamidino-2-phenylindole (DAPI) from Sigma-Aldrich (St. Louis, MO, USA); and anti-Myc antibody from Cell Signaling Technology (Danvers, MA, USA).

## Plasmid construction

3xPPRE-tk-Luc reporter plasmid was from Addgene, and renilla luciferase was purchased from Promega (Madison, WI, USA). Triple Flag tag (abbreviated as ‘Flag’ hereafter), triple Myc tag (as ‘Myc’ hereafter), turboRFP, or EGFP DNA sequences were tagged at the N-terminus of EGFP, PPARγ, HNF4α, or STK38 [19] by PCR using Phusion polymerase (New England Biolabs, Ipswich, MA) and cloned into a pENTR1A vector. For making shSTK38, primers 5ʹ-ACCTCGTTCATGCAGACCGTACAATCAAGAGTTGTACCCGGTCTGCATGAACTT-3ʹ (forward) and 5’-CAAAGTTCATGCAGACCGTACAACTCTTGATTGTACCCGGTCTGCATGAACG-3ʹ were annealed and cloned into a pENTR1A vector containing psiRNA-h7SK cassette (InvivoGen, San Diego, USA) by Gibson cloning. All inserts were validated without mutation by Sanger sequencing. Respective pENTRA vectors with inserts were converted into lentiviral pSMPUW vectors (Cell Biolabs, San Diego, CA, USA) [4] via Gateway cloning. The knockdown efficiency of the shSTK38 construct was more than 90% in pilot studies.

## Cell culture, lentivirus production, and PPARγ-interacting protein isolation and identification

HEK-293 (CRL-1573, ATCC, Manassas, VA) and HEK-293 T (CRL-3216, ATCC) cells were maintained in DMEM (Dulbecco’s modified Eagle’s medium) containing 10% foetal bovine serum (FBS, Gibco Life Technologies), 100 μg of penicillin/ml, and 100 μg of streptomycin/ml (Gibco Life Technologies) for plasmid transfection or viral production.

For lentivirus production, transfer vector was co-transfected with packaging vector pCD/NL-BH*DDD (Addgene#17,531) and envelope vector pCMV-VSVG (Cell Biolabs) using LipoD293 in HEK-293 T cells as previously described [4,23]. To isolate PPARγ-interacting proteins, roughly 5 × 10^7^ adipose stromal vascular cells [24,25], which had been tested to differentiate well into adipocytes with lentiviral PPARγ infection, were plated in DMEM containing 10% FBS in a 10 cm dish. At 70% confluency, the cells were transduced with lentiviruses expressing Flag-EGFP, Flag-PPARγ, or Flag-M3-PPARγ [4]. Twenty-four hours later, the cells were treated with rosiglitazone (1 µM) and insulin (70 nM) for an additional 24 h. Thereafter, the cells were washed with 1x PBS and lysed in binding buffer (50 mM Tris, pH 7.5, 150 mM NaCl, 1% NP 40, 0.25% sodium deoxycholate, 10% glycerol, 1 mM EGTA, 1 mM PMSF, protease inhibitor cocktail). The lysates were then centrifuged, and the supernatants were incubated with 20 ml of anti-Flag M2 magnetic beads overnight at 4°C. The beads were collected using a magnetic stand and thoroughly washed with washing buffer (250 mM NaCl). Binding proteins were eluted with 200 mM Flag peptide in washing buffer and subjected to proteomics analysis with LC (liquid chromatography)–tandem mass spectrometry at the Proteomics Core of the University of Maryland School of Medicine and reported as the number of unique peptides matching existing proteins.

## Immunoprecipitation and Western blot analyses

For immunoprecipitation experiments, HEK-293 T cells were grown in 15 cm dishes and transiently transfected with Flag- or Myc-tagged PPARγ, STK38, HNF4α, and EGFP expression vectors (10 μg) using LipoD293 (Signagen). Forty-eight hours post-transfection, the cells were lysed in RIPA buffer. Cell lysates were incubated with anti-Flag beads or with both anti-Myc antibody and protein A resin overnight at 4°C, washed, and dissolved in 1X sample buffer for Western blot analysis. We repeated the proteomics studies three times using preadipocytes from three subjects to avoid possible bias due to donor variation.

For Western blot analysis, cell lysates or immuno-precipitates were separated on SDS–polyacrylamide gel electrophoresis and then transferred to PVDF membranes. The blots were probed with anti-Flag or anti-Myc antibody and alkaline phosphatase (AP)-conjugated secondary antibodies, visualized by the BCIP/NBT Liquid Substrate System (Sigma-Aldrich) and quantified with ImageJ (Bethesda, MD).

## Protein co-localization and stability studies

HEK293 cells were co-transfected with plasmids overexpressing EGFP-STK38 (0.1 µg mg) and turboRFP-PPARγ (0.2 mg) in Nest Scientific 4-Well Cell Culture Chamber Slide (Stellar Scientific, Baltimore, Maryland). Forty-eight hours after transfection, the cells were fixed with 4% paraformaldehyde for 30 min at room temperature, then washed with PBS, and stained with DAPI (0.1 mg/ml) for 10 min. The slide was then washed, mounted with a coverslip, and imaged by a fluorescence microscope. For protein stability assays, HEK293 cells were co-transfected with 1 µg plasmid of Flag-, Myc-PPARγ with Myc-STK38 or Myc-HNF4α in a 6-well plate. Twenty-four hours post-transfection, the cells were treated with cycloheximide (300 μg/ml), and cell lysates were collected for Western blot analysis at the indicated time points following treatment.

## Adipogenesis and RT-qPCR

For adipocyte differentiation, preadipocytes were plated in duplicates, cultured in DMEM with 10% bovine serum, 100 μg of penicillin/ml, and 100 μg of streptomycin/ml, transduced with lentiviruses of EGFP, PPARγ, STK38, or shSTK38, and grown to confluency. The cells were then switched to differentiation medium containing dexamethasone (250 nM), 3-isobutyl-1-methylxanthine (500 μM), and insulin (170 nM) for 2 days. On day 3, the medium was changed to culture medium supplemented with insulin (170 nM) and left for 7 days. Subsequently, the cells were either stained with Oil-Red-O or collected for total RNA extraction. For Oil-Red-O staining, differentiated cells were fixed with 10% formalin at 4°C for 1 h, followed by 1x PBS wash and staining with 0.35% Oil-Red-O (Sigma) at 25°C for 1 h. Then, Oil-Red-O was washed away with distilled water, and the stained cells were imaged under a microscope, and isopropanol was added to elute Oil-Red-O. An aliquot of the eluate was then measured for absorbance at 510 nm by a microplate reader. For RT-qPCR, total RNAs were extracted using RNeasy Lipid Tissue Mini Kit (Qiagen, Valencia, CA) with on-column DNase digestion (Qiagen) as described before [25].cDNAs were synthesized using AMV Reverse Transcriptase kit (Promega) from 1 µg of total RNA. Quantitative PCR was performed on a Light Cycler 480 (Roche, Indianapolis, IN) using primers 5ʹ-acaccttgctcatccatgag-3ʹ (forward) and 5ʹ- ggccttcttcttccatcacc-3ʹ (reverse) for STK38, 5ʹ- cctttαaatactgagatttccttca -3ʹ (forward), 5ʹ- ggacacccccatctaaggtt-3ʹ (reverse) for FABP4, and 5ʹ- atgcactggaattagatgacag-3ʹ (forward) and 5ʹ- acaagtccttgtagatctcctg-3ʹ for PPARγ. Primers for FABP4 and γ- Actin were described previously [4]. Beta-actin mRNA was used for normalization of cDNA loading as an internal control. The relative expression of the target genes compared to beta-actin was determined by the 2-ΔΔCT method.

## Data analysis

Data were presented as mean ± SEM. One-way or two-way analysis of variance (ANOVA), followed by post-hoc Bonferroni’s test for between-group comparisons or by Dunnett’s test for test group comparisons with the control, was used to determine the group-to-group statistical significance. The half-life of protein degradation was calculated by using the equation of exponential one-phase decay. Statistical analyses were performed using GraphPad Prism 5. Differences were considered statistically significant at the level of *p* <0.05.

## Supplementary Material

Supplemental Material

## Data Availability

Kun Qian had full access to all the data in the study and take responsibility for the integrity of the data and the accuracy of the data analysis.

## References

[1] Wu Z, Puigserver P, Andersson U, et al. Mechanisms controlling mitochondrial biogenesis and respiration through the thermogenic coactivator PGC-1. Cell. 1999;98(1):115–124. 10412986 10.1016/S0092-8674(00)80611-X

[2] Zhu Y, Kan L, Qi C, et al. Isolation and characterization of peroxisome proliferator-activated receptor (PPAR) interacting protein (PRIP) as a coactivator for PPAR. J Biol Chem. 2000;275(18):13510–13516. 10788465 10.1074/jbc.275.18.13510

[3] Van Beekum O, Brenkman AB, Grontved L, et al. The adipogenic acetyltransferase Tip60 targets activation function 1 of peroxisome proliferator-activated receptor gamma. Endocrinology. 2008;149(4):1840–1849. 18096664 10.1210/en.2007-0977

[4] Zhu Y, Yang R, McLenithan J, et al. Direct conversion of human myoblasts into brown-like adipocytes by engineered super-active PPARγ. Obesity. 2015;23(12):1014–1021. 25919922 10.1002/oby.21062 PMC4413469

[5] Ge K, Cho YW, Guo H, et al. Alternative mechanisms by which mediator subunit MED1/TRAP220 regulates peroxisome proliferator-activated receptor gamma-stimulated adipogenesis and target gene expression. Mol Cell Biol. 2008;28(3):1081–1091. 18039840 10.1128/MCB.00967-07 PMC2223395

[6] Zhu Y, Qi C, Jain S, et al. Isolation and characterization of PBP, a protein that interacts with peroxisome proliferator-activated receptor. J Biol Chem. 1997;272(41):25500–25506. 9325263 10.1074/jbc.272.41.25500

[7] Tasdelen I, Van Beekum O, Gorbenko O, et al. The serine/threonine phosphatase PPM1B (PP2Cbeta) selectively modulates PPARγ activity. Biochem J. 2013;451(1):45–53. 23320500 10.1042/BJ20121113

[8] Katano-Toki A, Satoh T, Tomaru T, et al. THRAP3 interacts with HELZ2 and plays a novel role in adipocyte differentiation. Mol Endocrinol. 2013;27(5):769–780. 23525231 10.1210/me.2012-1332 PMC5416755

[9] Hergovich A, Schmitz D, Hemmings BA. The human tumour suppressor LATS1 is activated by human MOB1 at the membrane. Biochem Biophys Res Commun. 2006;345(1):50–58. 16674920 10.1016/j.bbrc.2006.03.244

[10] Bao Y, Sumita K, Kudo T, et al. Roles of mammalian sterile 20-like kinase 2-dependent phosphorylations of Mps one binder 1B in the activation of nuclear Dbf2-related kinases. Genes Cells. 2009;14(12):1369–1381. 19919647 10.1111/j.1365-2443.2009.01354.x

[11] Hergovich A. Regulation and functions of mammalian LATS/NDR kinases: looking beyond canonical Hippo signalling. Cell Biosci. 2013;3(1):32. 23985307 10.1186/2045-3701-3-32 PMC3849777

[12] Cornils H, Kohler RS, Hergovich A, et al. Human NDR kinases control G(1)/S cell cycle transition by directly regulating p21 stability. Mol Cell Biol. 2011;31(7):1382–1395. 21262772 10.1128/MCB.01216-10 PMC3135299

[13] Martin AP, Jacquemyn M, Lipecka J, et al. STK38 kinase acts as XPO1 gatekeeper regulating the nuclear export of autophagy proteins and other cargoes. EMBO Rep. 2019;20(11):e48150. 31544310 10.15252/embr.201948150 PMC6832005

[14] Floyd ZE, Stephens JM. Control of peroxisome proliferator-activated receptor gamma2 stability and activity by SUMOylation. Obesity Res. 2004;12(6):921–928. 10.1038/oby.2004.112 15229330

[15] Li P, Song Y, Zan W, et al. Lack of CUL4B in adipocytes promotes PPARγ-mediated adipose tissue expansion and insulin sensitivity. Diabetes. 2017;66(2):300–313. 27899484 10.2337/db16-0743

[16] Hauser S, Adelmant G, Sarraf P, et al. Degradation of the peroxisome proliferator-activated receptor gamma is linked to ligand-dependent activation. J Biol Chem. 2000;275(24):18527–18533. 10748014 10.1074/jbc.M001297200

[17] Tsai YC, Tsai SH, Chang EY, et al. Cytoskeletal protein vimentin interacts with and regulates peroxisome proliferator-activated receptor gamma via a proteasomal degradation process. J Cell Biochem. 2013;114(7):1559–1567. 23297177 10.1002/jcb.24497

[18] Grossman SR, Perez M, Kung AL, et al. p300/MDM2 complexes participate in MDM2-mediated p53 degradation. Mol Cell. 1998;2(4):405–415. 9809062 10.1016/s1097-2765(00)80140-9

[19] Bisikirska BC, Adam SJ, Alvarez MJ, et al. STK38 is a critical upstream regulator of MYC’s oncogenic activity in human B-cell lymphoma. Oncogene. 2013;32(45):5283–5291. 23178486 10.1038/onc.2012.543 PMC3715597

[20] Cao R, Wang G, Qian K, et al. TM4SF1 regulates apoptosis, cell cycle and ROS metabolism via the PPARγ-SIRT1 feedback loop in human bladder cancer cells. Cancer Lett. 2018;414:278–293. 29175458 10.1016/j.canlet.2017.11.015

[21] Wang G, Cao R, Wang Y, et al. Simvastatin induces cell cycle arrest and inhibits proliferation of bladder cancer cells via PPARγ signalling pathway. Sci Rep. 2016;6(1):35783. 27779188 10.1038/srep35783 PMC5078845

[22] Yang PL, Wang JS, Cheng XM, et al. PPAR-gamma ligand inhibits nasopharyngeal carcinoma cell proliferation and metastasis by regulating E2F2. PPAR Res. 2019;2019:8679271. 31467515 10.1155/2019/8679271 PMC6699258

[23] Wang Z, Yu D, Wang M, et al. Elabela-apelin receptor signaling pathway is functional in mammalian systems. Sci Rep. 2015;5(1):8170. 25639753 10.1038/srep08170 PMC4313117

[24] Yang RZ, Lee MJ, Hu H, et al. Acute-phase serum amyloid A: an inflammatory adipokine and potential link between obesity and its metabolic complications. PLoS Med. 2006;3(6):e287. 16737350 10.1371/journal.pmed.0030287 PMC1472697

[25] Yang RZ, Lee MJ, Hu H, et al. Identification of omentin as a novel depot-specific adipokine in human adipose tissue: possible role in modulating insulin action. Am J Physiol Endocrinol Metab. 2006;290(6):E1253–61. 16531507 10.1152/ajpendo.00572.2004

