# Supplementary material for: STK38 is a PPARγ-interacting protein promoting adipogenesis
Source: Adipocyte. 2021 Oct 20;10(1):524–31. doi: 10.1080/21623945.2021.1980257 (PMC8726646; doi:10.1080/21623945.2021.1980257)
Supplement: Supplemental Material [file KADI_A_1980257_SM8863.docx]

**STK38 is a PPARγ-interacting Protein Promoting Adipogenesis**

**Supplemental Data**

1. **Validation of STK38 suppression by shSTK38.**

Suppl. Fig 1. Validation of STK38 suppression by shSTK38. Plasmid EGFP-STK38 was transfected (+) either alone or with shSTK38 or scramble control construct in HEK293 cells for 48 hrs. The representative images of the cells under fluorescence microscopy are shown in the upper panel and western blotting for EGFP and β-actin is in the lower panel. The knockdown efficiency of EGFP-STK38 by shSTK38 was about 91%.

1. **Suppl. Table 1. Selected gene expression in preadipocytes and differentiated adipocytes.**

| Gene | Preadipocytes  (Mean ± SD) | Adipocytes  (Mean ± SD) | Ratio of Pre./Ad. |
| --- | --- | --- | --- |
| β-actin | 1872.15±355.41 | 2016.98±500.56 | 1.08 |
| GAPDH | 1577.09±519.04 | 2508.07±935.36 | 1.59 |
| STK38 | 2.85±0.71 | 5.50±2.06 | 1.93 |
| STK38L | 0.46±0.24 | 0.27±0.13 | 0.58 |

Human preadipocytes were subjected to adipogenic differentiation for 11 days. Poly(A) RNA was extracted from human preadipocytes (Pre.) and differentiated adipocytes (Ad.) for RNAseq analyses. Data are expressed as mean ± SD (n = 3) in RPKM (Reads per kilo base per million mapped reads).
